# The predominance of Human Immunodeficiency Virus type 1 (HIV-1) circulating recombinant form 02 (CRF02_AG) in West Central Africa may be related to its replicative fitness

**DOI:** 10.1186/1742-4690-3-40

**Published:** 2006-07-03

**Authors:** Harr F Njai, Youssef Gali, Guido Vanham, Claude Clybergh, Wim Jennes, Nicole Vidal, Christelle Butel, Eitel Mpoudi-Ngolle, Martine Peeters, Kevin K Ariën

**Affiliations:** 1 HIV and Retrovirology Research Unit, Department of Microbiology, Institute of Tropical Medicine, 155 Nationalestraat, B-2000 Antwerp, Belgium; 2 Department of Biomedical Sciences, Faculty of Pharmaceutical, Veterinary and Biomedical Sciences, University of Antwerp, Universiteitsplein 1, 2610 Antwerpen, Belgium; 3 Immunology Unit, Department of Microbiology, Institute of Tropical Medicine, 155 Nationalestraat, B-2000 Antwerp, Belgium; 4 Institut de Recherche pour le Développement (IRD-UR 36) and Department of International Health, University of Montpellier, Montpellier, France; 5 Projet Presica, Hopital Militaire de Yaounde, BP 906, Yaounde, Cameroon

## Abstract

**Background:**

CRF02_AG is the predominant HIV strain circulating in West and West Central Africa. The aim of this study was to test whether this predominance is associated with a higher in vitro replicative fitness relative to parental subtype A and G viruses. Primary HIV-1 isolates (10 CRF02_AG, 5 subtype A and 5 subtype G) were obtained from a well-described Cameroonian cohort. Growth competition experiments were carried out at equal multiplicity of infection in activated T cells and monocyte-derived dendritic cells (MO-DC) in parallel.

**Results:**

Dual infection/competition experiments in activated T cells clearly indicated that CRF02_AG isolates had a significant replication advantage over the subtype A and subtype G viruses. The higher fitness of CRF02_AG was evident for isolates from patients with CD4+ T cell counts >200 cells/μL (non-AIDS) or CD4+ T cell counts <200 cells/μL (AIDS), and was independent of the co-receptor tropism. In MO-DC cultures, CRF02_AG isolates showed a slightly but not significantly higher replication advantage compared to subtype A or G isolates.

**Conclusion:**

We observed a higher *ex vivo *replicative fitness of CRF02_AG isolates compared to subtype A and G viruses from the same geographic region and showed that this was independent of the co-receptor tropism and irrespective of high or low CD4+ T cell count. This advantage in replicative fitness may contribute to the dominant spread of CRF02_AG over A and G subtypes in West and West Central Africa.

## Background

Mutation and recombination are important mechanisms by which HIV evades host immune responses and antiretroviral drug pressure [1]. Recombinant strains of HIV-1 have been found worldwide [2-8]. To date, sixteen Circulating Recombinant Forms (CRFs) have been characterized according to the Los Alamos HIV sequence database and at least two are of major epidemiological importance. CRF01_AE [2,3,5] and CRF02_AG [6,7] are causing heterosexual epidemics in Asia and West and West Central Africa, respectively. CRF02_AG caused approximately 5.3% of all new HIV-infections globally between 1998 and 2000, but is responsible for nearly 31% of new infections in West Africa and about 6.7% in Central Africa [8–10, UNAIDS]. Earlier studies with smaller numbers of samples and originating from various African countries consistently showed that CRF02_AG is more prevalent than HIV-1 subtypes A and G in West and Central Africa [10-15]. In the mean time, CRF02_AG viruses have been introduced in Europe and, to a minor extent, in the US and Puerto Rico [16,17].

In West and West Central Africa HIV types (1 and 2), HIV-1 groups (M, O, N) and many subtypes co-circulate [18,19]. Cameroon, a country in West Central Africa, has the genetically most diverse HIV epidemic in the world and the wide variety of co-circulating HIV groups and subtypes are a major source for intersubtype recombinants (ISRs) and CRFs [20]. Interestingly, prevalence rates for CRF02_AG seem to increase more rapidly than prevalence rates of other subtypes in West Africa and suggest that, particularly in Cameroon, CRF02_AG may spread more rapidly than other clades [21-23]. The emergence of CRF02_AG as the predominant strain causing HIV infections in West Africa may simply be the result of a founder effect. However, theoretically genetic recombination and selection may combine the best characteristics of two (or more) viruses and as such provide an advantage to the recombinant over other strains. This raises concern that CRF02_AG may be favored, in terms of a superior replicative fitness and/or transmission efficiency, over other co-circulating strains.

Several studies relate the differential spread of HIV-1 group M, group O and HIV-2 in the human population (i.e. *in vivo *fitness) to differences in transmission [24,25] and pathogenesis [26]. Recent findings on the *in vitro *replicative fitness of diverse human immunodeficiency viruses support the hypothesis that the relative replicative fitness and the prevalence of viral types and subtypes are related. It was shown that HIV-1 group O and HIV-2 primary isolates had a reduced fitness in activated T cells and in dendritic cells as compared to HIV-1 group M primary isolates of subtypes A, B, C, D and CRF01_AE, corroborating with the much higher prevalence of group M, as compared to group O and HIV-2 in the pandemic [27]. Furthermore, lower replicative fitness of HIV-2 isolates compared to HIV-1 group M viruses could be related to the delayed disease progression observed with HIV-2 infections [27].

In the present study, we tested whether the *ex vivo *replicative fitness of CRF02_AG may be related to its predominance in West Central Africa. Therefore, we performed pair-wise competitions using a number of primary CRF02_AG strains and primary subtype A and G viruses, all sampled in Cameroon. In order to mimic two relevant micro-environments, we performed viral competitions in activated T cells and in dendritic cells (DC). Activated T cells are the major source of circulating HIV *in vivo*. For *in vitro *testing, activated T cells can easily be generated by mitogen stimulation of peripheral blood mononuclear cells (PBMC). Although primary DC are more difficult to obtain, monocyte-derived dendritic cells (MO-DC) can be generated abundantly and have an interstitial-like phenotype (i.e. DC-SIGN+, CCR5+, high T cell stimulatory capacity) which makes them a representative model for DC in the genital mucosae. These cells are thought to have a crucial role in the early events of heterosexual HIV transmission [28,29].

## Results

### Characterization of primary HIV-1 isolates

**Figure 1:**
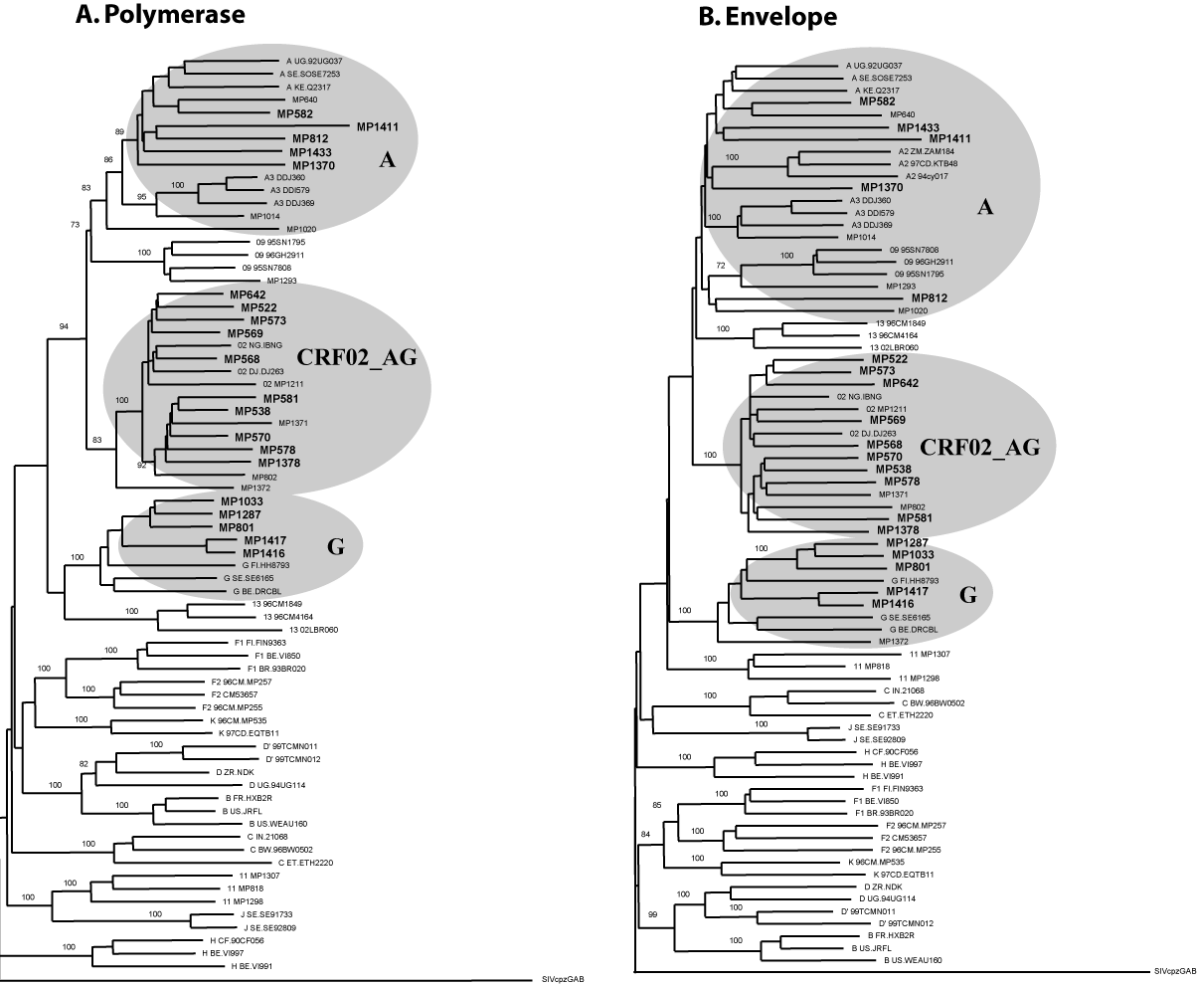
**Virus phylogeny**. The complete *env *and *pol *coding regions were sequenced for each virus isolate (EMBL accession numbers: *env*; AM279343–AM279369 and *pol*; AM279370–AM279396). Subsequently, NJ-trees were constructed and tree topology was assessed by bootstrap analysis. The SIV_cpzGAB _sequence was used to root the tree. Ten isolates were found to group with the CRF02_AG reference strains, five were subtype A and five were subtype G.

Twenty HIV-1 isolates were obtained from a patient cohort in Cameroon, previously described by Laurent *et al*. [14]. Fifteen isolates were found to use only CCR5, while three viruses could use only CXCR4 and two others were able to use both CCR5 and CXCR4 as entry co-receptor (Table 1). Sequencing and subsequent phylogenetic analysis of the complete *env *and *pol *regions, *gag *p24 and p17 regions, and accessory genes (*tat, rev, nef, vpu*) revealed that ten isolates were CRF02_AG, five were subtype A and five were subtype G (Table 1, Figure 1). CD4+ T cell counts in this patient cohort showed a wide variation (from 0 to >1000 cells/μl blood). We subdivided the patients according to their CD4+ T cell count, i.e. twelve samples with >200 cells/μl and eight samples with <200 cells/μL (AIDS) (Table 1). Plasma viral load was measured for each patient at the time of virus isolation. In concordance with recent observations by Fischetti *et al*. [21] and Sarr *et al*. [23], we observed an overall trend to slightly higher viral load in a random sample of individuals infected with CRF02_AG (average VL_(CRF02_AG)} _= 5.13 Log10 RNA copies/mL), compared to those infected with a subtype A or G isolate (average VL_(subtype A and G) _= 4.58 Log10 RNA copies/mL) (Table 1), although this difference was not statistically significant (P = 0.213, t-test). Furthermore, individuals infected with CRF02_AG appeared to have reduced peripheral CD4+ T cell counts compared to subjects infected with a subtype A or G virus (average CD4+ T cell count_(CRF02_AG) _= 226 cells/μL and average CD4+ T cell count_(Subtype A and G) _= 334 cells/μL), but again not statistically significant (P = 0.379, t-test). The samples were randomly selected from an African patient cohort and there was no data available on the duration of the infection, or on the precise clinical condition, but obviously there may be considerable difference in the stage of disease at which these viruses were isolated. Interestingly, previous studies have shown that the replicative fitness of HIV-1 correlates with disease progression [30,31]. Therefore, we have analyzed the relative viral fitness of samples from infected subjects with CD4+ T cell counts above and below 200 cells per microliter, separately.

**Table 1:** Virus characteristics. Virus and patient characteristics of primary HIV-1 isolates obtained from Cameroon. Subtyping was based on complete *pol *and complete *env*, *gag *p24, *gag *p17, *tat*, *rev*, *nef*, and *vpu *nucleotide sequences. CD4+ T cell counts and viral load were determined at the time of virus isolation. Co-receptor usage was tested on U87.CD4 cells expressing either CCR5 or CXCR4.

**Virus Isolate**	**Subtype**				**Year of isolation**	**Country**	**CD4+ T cell count**^9^	**Viral load**^10^	**Co-receptor tropism**
					
	*env*^1^	*pol*^2^	*gag*^3^	other^4^					
						
MP569	AG	AG	AG^5^	-	1997	CM	1029	2.95	R5
MP538	AG	AG	AG^5^	-	1996	CM	350	4.49	R5
MP573	AG	AG	AG^5^	-	1997	CM	277	5.67	R5
MP568	AG	AG	AG^5^	-	1997	CM	266	4.91	R5
MP570	AG	AG	AG^5^	-	1997	CM	213	5.41	R5
								
						Average	427	4.69	
									
MP642	AG	AG	AG	AG^7^	1997	CM	104	5.71	R5
MP578	AG	AG	AG	AG^7^	1997	CM	8	5.41	R5X4
MP581	AG	AG	AG^5^	-	1997	CM	8	5.80	X4
MP522	AG	AG	AG^5^	-	1996	CM	2	5.10	X4
MP1378	AG	AG	AG^5^	AG^8^	1999	CM	0	5.89	R5
								
						Average	24	5.58	
									
MP801	G	G	G^6^	G^8^	1997	CM	731	3.85	R5
MP582	A	A	A^5^	A^7^	1997	CM	521	3.78	R5
MP1370	A	A	-	-	1999	CM	477	4.31	R5
MP1033	G	G	G^6^	G^8^	1998	CM	394	2.39	R5
MP1416	G	G	G	G^7^	1999	CM	368	5.59	R5
MP1433	A	A	-	-	1999	CM	321	4.93	R5
MP812	A	A	A^5^	A^8^	1997	CM	310	4.52	X4
								
						Average	446	4.20	
									
MP1411	A	A	-	-	1999	CM	105	5.18	R5
MP1287	G	G	-	-	1999	CM	91	5.83	R5
MP1416	G	G	G	G^7^	1999	CM	23	5.38	R5X4
								
						Average	73	5.46	

^1^Complete env nucleotide sequence

^2^Complete pol nucleotide sequence

^3^Gag p24 and gag p17 nucleotide sequence

^4^Accessory gene nucleotide sequence (tat, rev, nef, vpu)

^5^Gag p24 nucleotide sequence only

^6^Gag p17 nucleotide sequence only

^7^Tat, rev, nef nucleotide sequence

^8^Vpu nucleotide sequence

^9^Cells/μl blood

^10^Log10 RNA copies/ml plasma

### Replicative fitness of CRF02_AG in activated human T-cells

**Figure 2:**
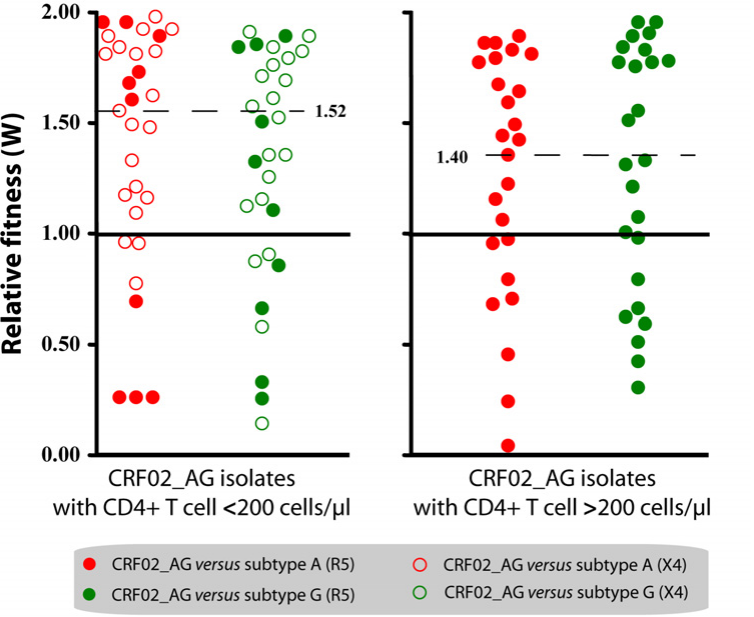
**Relative replicative fitness (W) in activated T cells**. Dot plots represent the results of growth competitions in PHA/IL-2 activated PBMC (10 CRF02_AG, 5 subtype A and 5 subtype G). Red dots represent competitions between CRF02_AG and subtype A viruses; green dots show competitions between CRF02_AG and subtype G. An open symbol indicates that the CRF02_AG virus is X4-tropic, whereas solid symbols represent competitions with R5-tropic CRF02_AG viruses (irrespective of the coreceptor tropism of the subtype A and G isolates). The competitions with CRF02_AG viruses from patients with AIDS (CD4+ cells <200 cells/μl) are shown at the left hand side and the competitions with CRF02_AG viruses from non-AIDS patients at the right hand side, again irrespective of the CD4+ T cell counts in the patients from which the competing A or G virus was derived.

Ten CRF02_AG were competed in duplicate against five subtype A and five subtype G isolates. CRF02_AG isolates won 68 out of 100 competitions (68%), resulting in a median relative fitness (W) of 1.50 (p25 = 0.96, p75 = 1.82). Subtype A isolates and subtype G viruses had a median relative fitness of 0.50 (p25 = 0.18, p75 = 1.03) and 0.66 (p25 = 0.22, p75 = 1.13), respectively. The median relative fitness values for CRF02_AG viruses were significantly higher than 1.0 (P < 0.001, t-test), and the relative fitness of both subtype A and G isolates were significantly lower than 1.0 (P < 0.001, t-test) (Figure 2) (with W = 1.0 being equal replicative fitness).

Earlier experiments showed that HIV-1 replicative fitness correlates directly with viral load and inversely with CD4+ T cell count [30,31]. Since, the CD4+ T cell counts and viral loads tended to differ between CRF02_AG and non-CRF02_AG infected subjects in our study population, we re-analyzed competitions of viral isolates obtained from CRF02_AG patients with CD4+ T cell counts **<**200 cells/μl and CD4+T cell counts **>**200 cells/μl against the entire set of subtype A and G viruses, irrespective of the CD4+ T cell counts in the patients from whom these viruses were isolated. In the group with CD4+ T cells <200 cells/μl, the CRF02_AG isolates won 35 out of 50 competitions (70%), with a median relative fitness of 1.52 (p25 = 1.07, p75 = 1.83). In the group with CD4+ T cells >200 cells/μl, the CRF02_AG isolates won 34 out of 50 (68.0%) of the competitions with a median relative fitness of 1.40 (p25 = 0.84, p75 = 1.79) (Figure 2). These observations suggest that the difference in replicative fitness between these viruses is not merely associated with the differences in CD4+ T cell counts and VL.

There is evidence that the co-receptor tropism may influence HIV-1 replication in T cells [30,32] and that syncytium-inducing (SI)/X4 viruses tend to be more virulent than NSI/R5 strains. Since our cohort consisted of both X4 and R5 tropic isolates, we analyzed the data correcting for viral co-receptor tropism. The majority of viruses were R5-tropic, three viruses used CXCR4 (X4), and two others were found to be dual-tropic (R5X4). The X4 CRF02_AG isolates won 6 out of 6 (100%) competitions against X4 subtype A and G viruses. Similarly, X4 CRF02_AG strains won 17 out of 24 (70.8%) competitions against R5 subtype A and G viruses. Interestingly, R5 CRF02_AG viruses also out competed most of the X4 subtype A and G strains (11 out of 14 competitions or 78.6%). Finally, R5 CRF02_AG won 62.5% (35 out of 56) competitions against the R5 subtype A and R5 subtype G.

These results suggest that the increased fitness of CRF02_AG in competitions with subtype A and G viruses is not caused by differential co-receptor tropism.

### Replicative fitness of CRF02_AG in dendritic cells

Since mucosal dendritic cells are thought to play an important role in the early phase of sexual transmission [28,29], assessing the replicative capacity of CRF02_AG and subtype A and G viruses in a suitable model of mucosa-like DC, such as the monocyte-derived DC, would allow us to study the replication efficiency of primary HIV isolates in the context of virus transmission. Because R5 viruses are consistently found early after transmission, we restricted our analysis to isolates of this phenotype (i.e. four CRF02_AG, one subtype A and three subtype G isolates).

**Figure 3:**
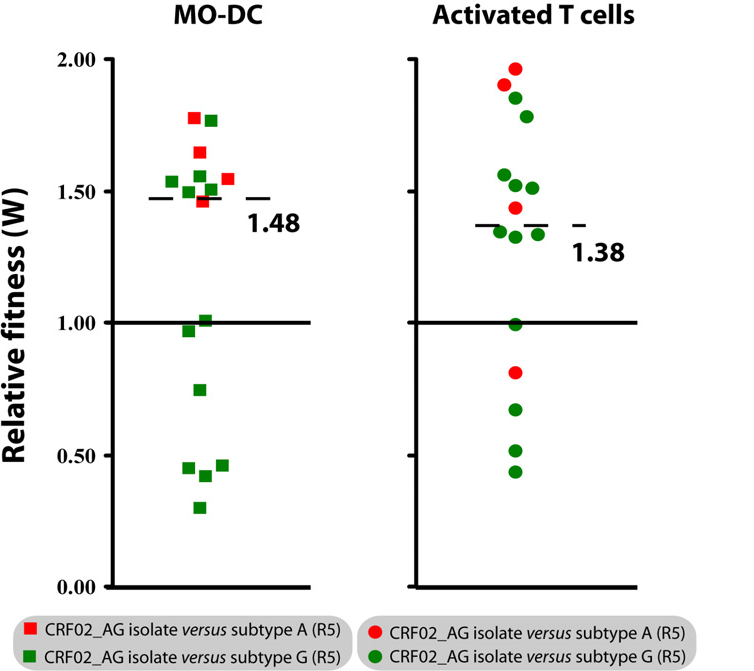
**Relative replicative fitness (W) in monocyte-derived dendritic cells (MO-DC)**: Dot plots represent growth competitions in MO-DC and activated T cells using the same R5-tropic viruses isolates (4 CRF02_AG, 1 subtype A and 3 subtype G isolates). Solid red squares indicate competitions of a CRF02_AG against an A virus in MO-DC and solid green squares indicate competitions of CRF02_AG against a G virus in MO-DC. Solid red circles represent competitions of a CRF02_AG against a subtype A virus in T cells and solid green circles show competitions of a CRF02_AG against a subtype G virus in T cells.

In MO-DC, CRF02_AG isolates won 62.5% (10 out of 16) of the competions and showed a median relative fitness of 1.48 (p25 = 0.68, p75 = 1.55, Figure 3). When comparing fitness data obtained in T-cells and DC, it was obvious that most of the CRF02_AG isolates that were able to out compete subtype A and G viruses in DC also out competed subtype A and G strains in T cells. In conclusion, we found that the replicative fitness of CRF02_AG and subtype A and G viruses is significantly different when measured in activated T cells (P = 0.024, t-test) and also tends to be different in DC, without reaching statistical significance (P = 0.229, t-test) (Figure 3).

## Discussion

The fact that CRF02_AG seems largely predominant over other circulating HIV strains in an African area with extremely high HIV genetic diversity may have several explanations. First, the recombinant form may have some biological advantage over the parental strains, including a possibly higher replicative fitness and/or transmission capacity. Second, the recombinant strain could have been introduced first in that particular area and consequently get established in a population before other subtypes entered the scene (founder-effect) [31]. In the case of the epidemiological spread of CRF02_AG in West Central Africa, the founder hypothesis is probably a less likely explanation. Several studies on the prevalence of HIV-1 subtypes in the Democratic Republic of Congo (DRC) have shown that subtypes A and G are relatively prevalent in this area [34-37]. Moreover, it is likely that at least a limited spread of subtype A and G viruses must have preceded the creation and spread of CRF02_AG in West Central Africa [35].

In the present study, we explored whether the replicative fitness of CRF02_AG was related to the epidemiological spread of this virus in extended areas of West and Central Africa. We showed that CRF02_AG primary isolates had a higher replicative fitness compared to subtype A and G isolates, in a cellular model for HIV pathogenesis (i.e. activated T-cells) and HIV transmission (i.e. MO-DC). The higher relative replicative fitness of CRF02_AG viruses was evident for isolates from patients with low (<200 cells/μL) and with higher (>200 cells/μL) CD4+ T cell counts, and it was found to be independent of the viral co-receptor use. An independent study investigating the same hypothesis was published recently and also showed an increased replicative capacity of CRF02_AG viruses compared to subtype A and G isolates, using basic virus growth kinetics as a measure of replication capacity [38]. In contrast to our study, Konings *et al*. [38] studied only thirteen HIV-1 isolates and presented limited data on viral load and CD4+ T cell counts. Furthermore, the growth competition assays used in our study are able to discriminate minor differences in replication capacity and also provide the internal control lacking in monoinfection kinetic assays, as used by Konings *et al*. [38-40].

The viral load in the donor and the integrity of mucosal tissues in the acceptor are amongst the most important determinants upon HIV transmission [41,42]. Previous observations by Fischetti *et al*. [21], showed significantly higher viral loads in asymptomatic CRF02_AG infected individuals compared to patients infected with non-CRF02_AG strains. A direct correlation between viral load and replicative capacity in activated T cells was repeatedly shown [30,31]. Taken together with our observations, these data suggest that patients infected with CRF02_AG strains may more easily transmit virus, because of a higher viraemia, which could be a consequence of the higher replicative fitness in activated T cells. This interpretation is consistent with the observation by Ariën *et al*. [27], who previously showed that group M viruses in general have a much higher *in vitro *replicative fitness than group O or HIV-2 viruses, corresponding to the relative spread of these viruses in the pandemic as a whole and in West Africa (where they all co-circulate) in particular.

One could argue that the observed higher relative fitness of CRF02_AG strains versus subtype A and G isolates in the present study simply reflects a more advanced disease stage of patients infected with CRF02_AG or a differential viral co-receptor tropism. However, we have shown that CRF02_AG with either X4 or R5 co-receptor tropism and derived from patients with more or less advanced disease (based on CD4+ T cell count) are on average more fit than subtype A or G viruses (Figure 2). In addition, our data suggests that the replicative fitness of CRF02_AG in MO-DC was slightly, but not significantly higher than the parental subtypes (A and G) (Figure 3). There is substantial evidence that DC play an important role during HIV transmission and it could be speculated that a slight advantage in replicative fitness in dendritic cells may have an important impact on transmission at the population level. On the other hand, the number of competitions performed in DC may just have been too low to result in a significant difference.

Studies by Ball *et al*. [43] and Ariën *et al*. [27] showed that viruses of subtypes B and C were equally fit in Langerhans' dendritic cells, while subtype C isolates were out competed by any other group M virus in activated PBMC. It is not completely clear yet how HIV replicative fitness in T cells and dendritic cells relate to transmission and epidemiological spreading. It is also possible that the focus on replicative capacity in DC as a measure of transmission efficiency may be too limited, since other cell types at the mucosal interface are likely involved in transmitting HIV. Hence a better model to study HIV transmission is desirable and should include Female Genital Tract (FGT) epithelia and other important target cells, such as T cells and macrophages, in addition to DC [41,42]. We are currently elaborating on such models in order to study early events during HIV transmission and replicative fitness.

The CRF02_AG genome is a mosaic of subtype A (*gag*, *vpr *and parts of *pol*, *env *and *nef*) and subtype G (*LTR*, *rev*, *tat *and parts of *pol*, *env*, and *nef*). An important question that needs to be answered is which part of the viral genome may be responsible for the increased replicative fitness of CRF02_AG. Unfortunately, our experimental set up did not allow us to study the contribution of individual genes to the overall replicative fitness of a virus isolate. Therefore, future studies should try to elucidate the role of those genes that have a mosaic appearance for their impact on the fitness of the recombinant virus. It is clear that recombination occurs often in dual- or super infected individuals, generating ISR. It could be speculated that those ISR that generate viable progeny subsequently undergo severe selection pressure by the innate and adaptive host immune responses and that only the most successful/fit ISR may eventually be able to spread epidemically and become a CRF.

More detailed analyses of HIV samples from West Africa have shown that CRF02_AG has already undergone further recombination [34,44]. Clearly, viral recombination is inevitable with the continuous intermixing of HIV subtypes and will have its impact on the evolution of the HIV epidemic. It is important to envisage that a CRF that we label as very fit today may be out competed by a new and even more fit recombinant virus tomorrow.

In conclusion, our data on a small, but carefully selected sample from a Cameroonian cohort clearly suggests that the prevailing CRF02_AG recombinant may be favoured in his spread over "parental" subtype A and G viruses as a result of a higher replicative fitness in T cells and likely also in dendritic cells. More extensive and in-depth studies are needed to confirm this preliminary evidence and to unravel the molecular mechanisms underlying the predominance of CRF02_AG in large parts of West Africa.

## Methods

### Cells

Peripheral blood mononuclear cells (PBMC) were obtained from a HIV-1 seronegative buffy coat by Ficol Hypaque (Sigma, St. Louis, USA) density gradient centrifugation. PBMC were cultured in RPMI 1640 – 2 mM L-glutamine medium (BioWhittaker, Verviers, Belgium) supplemented with 10% fetal bovine serum (Biochrom KG, Berlin, Germany) and 100 U/ml penicillin (Cellgro, Virginia, USA) and 100μg/ml streptomycin (Cellgro, Virginia, USA). They were first stimulated with 2μg/ml of phytohemagglutinin (PHA) (Gibco BRL, Maryland USA) for 3 days and further maintained in 1 ng/ml interleukin (IL-2) (Gibco BRL, Maryland USA).

Monocytes were obtained from PBMC by counter-flow elutriation and sheep erythrocyte rosetting, yielding >95% CD3-/CD4+ MO and <0.5% T cells, as previously described in [28] and [45]. To obtain MO-DC, monocytes were cultured for 7 days in RPMI 1640 supplemented with 10% FBS, IL4 (Gibco BRL, Maryland USA) (20 ng/ml), GM-CSF (Gibco BRL, Maryland USA) (20 ng/ml), 100 U/ml penicillin and 100μg streptomycin [28,45]. Half of the culture medium (with cytokines) was replaced every third day. The MO-DC were immuno phenotyped as CD13^+^/CD14 low, CD3^-^/CD4^+^, CD1a^+ ^and DC-SIGN high before use.

### Viruses

Twenty viruses were obtained from HIV seropositive patients attending the military hospital in Yaounde and Douala in Cameroon [14]. Viruses were isolated between 1996 and 1999 and none of the patients was receiving antiretroviral treatment (ART) at that time. All patients signed an individual informed consent. We selected these twenty strains from a much larger cohort [14], based on the availability of PBMC and plasma, simultaneously obtained from these particular patients and permanently frozen in liquid nitrogen and at -80°C, respectively. CD4+ T cell counts were determined on fresh blood, while viral load was measured on the stored plasma samples for the purpose of this study, using an in-house real time PCR assay (Table 1). The original selection encompassed twenty-seven isolates (eleven CRF02_AG, ten subtype A and six subtype G), but six samples were dropped for further analyses because they showed unique recombination events to have occurred in *env *and *pol*, i.e. they were not pure A, nor G, nor CRF02_AG. For the twenty primary isolates used in this study, subtyping was based on complete *env*, complete *pol*, *gag *p24, *gag *p17, *tat*, *rev*, *nef *and *vpu *nucleotide sequencing.

Frozen virus stocks were propagated and expanded in short-term cultures of PHA/IL-2 treated PBMC obtained from a HIV seronegative blood donor. The 50% tissue culture infectious dose (TCID_50_) was determined by serial dilution of the virus stock to infect PHA/IL-2 PBMC and U87.CD4 cells expressing either CCR5 or CXCR4 (Table 1) [46]. Infections with U87.CD4.CCR5 and U87.CD4.CXCR4 cells were used to determine co-receptor tropism and to calculate the infectious dose required to infect MO-DC.

### Sequencing and phylogenetic analysis

The HIV-1 strains characterized in this study were cultured in patient peripheral blood mononuclear cells. DNA was then extracted from the infected cells using the Qiagen DNA isolation kit (Qiagen S.A., Courtabeauf, France). Complete sequences for the *pol *and the *env *genes were generated. The first fragment, spanning the *gag-pol *region, was amplified with G00 (5'-GACTAGCGGAGGCTAGAAG-3', position 761–780 on HxB2) and HPOL4538 (5'-TACTGCCCCTTCACCTTTCCA-3', position 4994–4973 on HxB2) as outer primers. A second round fragment was obtained from a hemi-nested PCR reaction with G25reverse (5'-GCAAGTGTTTTGGCTGAAGCAAT-3', position 1872–1895 on HxB2) and HPOL4538. The second fragment, covering the accessory genes *tat*, *rev *and *nef*, was amplified using HPOL4235 (5'-CCCTACAATCCCCAAAGTCAAGG-3', position 4668–4691 on HxB2) and LSIGI (5'-TCAAGGCAAGCTTTATTGAGGCTTAAGCAG-3', positions 9647-9617/542-512 on HxB2). A second round fragment was then generated with envB (5'-AGAAAGAGCAGAAGACAGTGGCAATGA-3', position 6216–6243 on HxB2) and envM (5'-TAGCCCTTCCAGTCCCCCCTTTTCTTTTA-3', position 9116–9087 on HxB2). Taq Expand Long Template PCR was used according to manufacturer's instructions (Roche, Indianapolis, USA). And with the following cycling conditions: 3 minutes denaturation at 92°C, 16 cycles at 92°C for 20 seconds, 50°C for 30 seconds and 68°C for 4 minutes, followed by 16 cycles with 20 second-increments at the elongation step and a final extension of 10 minutes. The amplified fragments were purified using a QiaQuick gel extraction kit (QIAGEN S.A., France), and then directly sequenced with primers encompassing the *pol *and the *env *regions by using Big-Dye Chemistry (Applied Biosystems, France) according to the instructions of the manufacturer. Electrophoresis and data collection were done on an Applied Biosystems 3100 Genetic Analyzer. The electropherogram plots were visualized and processed under DNASTAR to generate consensus from the different overlapping sequences.

The newly determined sequences were aligned with known representatives of the different subtypes, sub-subtypes and CRFs described in Africa, using Clustal W. Sites with any gap between the sequences and areas of uncertain alignment were excluded from the analysis. Pair wise evolutionary distances were estimated with Kimura's two parameters method. Phylogenetic trees were constructed by NJ method, and the reliability of the tree topology was assessed by bootstrap analysis. Simplot 3.2 beta software (Stuart Ray, ), was used to investigate the recombinant structure of the newly sequenced genes. Similarity and bootscan plots were performed as already described. Briefly, similarity plots determined the percent similarity between a newly determined sequence and selected groups of references, by moving a window of 400 base pairs with 20 base pairs increments along the genome alignment. Similarity values were plotted at the midpoint of the 400 base pairs fragment. For the bootstrap plots, the SimPlot software performed bootscanning on neighbor joining trees by using SEQBOOT, DNADIST (with Kimura two parameters method and F84 model of maximum likelihood method, transition/transversion ratio = 2.0), NEIGHBOR and CONSENSE from the PHYLIP package for a 400 base pairs window moved along the alignment in increments of 20 base pairs. One thousand bootstraps replicates were evaluated for each phylogeny. The bootstrap values for the studied sequences were plotted at the midpoint of each window. In these two sets of analyses, the new sequences were compared with consensus sequences (50% threshold) representing the different HIV-1 clades from the same alignment used for phylogenetic tree analysis. Finally, all nucleotide sequences were submitted to the EMBL Nucleotide Sequence Database (accession numbers: *env*; AM279343–AM279369 and *pol*; AM279370–AM279396).

### Dual infection/competition assays

Dual infection/competition experiments were performed as previously described [27,30-32]. In short, all CRF02_AG were competed against 5 subtype A and 5 subtype G, (Table 1) in PHA/IL-2PBMC from one donor in 24 well culture plates and in duplicate. It is important to mention that aliquots of PBMC of the same buffy coat were used to grow the virus stocks, determine infectious titers and perform the competitions. A second set of competitions was performed using all available NSI/R5 isolates in MO-DC from another donor. In these competition experiments, cells (2 × 10^5 ^PHA/IL-2 PBMC or 1 × 10^6 ^MO-DC) were infected with two isolates at equal multiplicity of infection (5 × 10^-4 ^MOI for PHA/IL-2 PBMC or 1 × 10^-3 ^MOI for MO-DCs) [27,32]. The estimated frequency of in vitro recombination between HIV isolates in the dual infections was less than 0.1%/1000 bp or well below the limit of HTA detection [30,40]. Uninfected cells were used as HIV-negative controls and mono-infected cultures of each virus were used as positive controls. Infected cell cultures were incubated at 37^°^C and 5% CO_2 _for 24 hours after which residual virus was washed away with 1x phosphate-buffered saline pH 7.4 (PBS). Infected cells were re-suspended in medium containing IL-2 (in case of PHA/IL-2 PBMC) or medium without IL-2 (for MO-DCs) and kept at 37^°^C and 5% CO_2 _for 14 days. Half the culture medium was replaced twice a week. Cell free supernatant were collected at day 7, 10 and 14 and analyzed for gag p24 content using an in-house p24 ELISA assay [47]. Cells were harvested at peak vireamia and stored at -80°C for subsequent analysis. A more detailed description of the dual/infection competition assay can be found in [39,40].

### Heteroduplex tracking assay

Genomic DNA was extracted from lysed PHA/IL-2PBMC using the QIAamp DNA blood kit (Qiagen). Viral DNA was PCR amplified using a set of external primers (envB; 5'-AGAAAGAGCAGAAGACAGTGGCAATGA-3' and ED14; 5'-TCTTGCCTGGAGCTGTTTGATGCCCCAGAC-3') and nested primers E80 (5'-CCAATTCCCATACATTATTGTC-3') and E125 (5'-CAATTTCTGGGTCCCCTCCTGAGG-3') to produce a ± 480 bp fragment, encoding the C2–C4 *env *region [30]. Both first round and second round PCR amplifications were carried out in 100μl reaction mixture under defined cycling conditions [30]. Subsequently, heteroduplex tracking assays (HTA) were preformed to estimate the amount of virus produced by each isolate in the competition, relative to the amount of virus produced in monoinfections [30]. The same genomic region of two subtype B HIV-1 strains (i.e. VI969-6 and JR-FL) was amplified and used as probes in the HTA. Probes were generated in amplification reactions using [γ-^32^P] ATP labeled E80 primer radiolabelled PCR-amplified probes were separated on 1% agarose gels and then purified using the QIAquick gel extraction kit (Qiagen). Reaction mixtures containing DNA annealing buffer (100 mM NaCl, 10 mm tris-HCl [pH 7.8], 2 mM EDTA, 10 μl of unlabelled PCR-amplified DNA from the competition cultures and approximately 0.1 pmol of radioactive probe DNA. Each dual infection/competition was analyzed in at least two independent HTA reactions using two radiolabeled probes. PCR amplicon and probe were denatured at 95°C for 3 min, 37°C for 5 min and then rapidly annealed on wet ice. After 30 minutes, DNA heteroduplexes were resolved on 5% TBE non-denaturing polyacrylamide (PAGE) gels (Bio-Rad) (75 min. at 200 V). Gels were then dried for 45 minutes at 80°C and exposed on a phosphor imaging screen overnight. Images were captured with a phosphor imager (Cyclone, PerkinElmer) and analysed with the OptiQuant software package (PerkinElmer).

### Estimation of relative viral fitness

Relative virus production (*ws*) of each isolate in a dual infection was calculated by dividing the amount of isolate DNA in the dual infection and the amount of the same isolate DNA in a monoinfection (as determined with the phosphor imager). From these *ws *values, relative fitness (*W*) values for each virus were obtained using the formula [*W = (ws1/(ws1 + ws2)) *x *2*], where *ws1 *and *ws2 *are relative virus production of isolate 1 and 2, respectively [30,40].

### Statistics

Average CD4+ T cell counts and average viral loads were calculated for each group of viruses (i.e. CRF02_AG and non-CRF02_AG). One sample t-tests were used to calculate whether differences in CD4+ T cell counts and VL between virus groups (i.e. CRF02_AG and non-CRF02_AG) were statistically significant.

Average and interquartile relative fitness values (W) were calculated for competitions involving CRF02_AG, subtype A and subtype G virus isolates. One sample t-tests was used to determine whether the relative fitness of a group of viruses (i.e. CRF02_AG, or subtype A, or subtype G) was significantly different from W = 1.0 (with W = 1.0 meaning equal relative fitness). For all analyses, the level of significance was set at P = 0.05.

## Authors' contributions

HFN *has performed the majority of the experimental work and data analysis, and has drafted the manuscript*.

YG *has contributed to the experimental work*.

GV *has contributed to the study-design and helped to draft the manuscript*.

CC *has contributed to the experimental work*.

WJ *has contributed to the data analysis*.

NV *has contributed to the experimental work (phylogenetic analysis)*.

CB *has contributed to the experimental work (sequencing)*.

EMN *is coordinator of Projet PRESICA and of the patient cohort*.

MP *has contributed to the study-design and provided the HIV-isolates*.

KKA *has contributed to the study-design and helped to draft the manuscript*.

All authors read and approved the final manuscript.

## Competing interests statement

The author(s) declare that they have no competing interests.
